# Electrospun Fibers Encapsulating *Triticum vulgare* Extract as a Potential Scaffold for the Regeneration of Subepithelial Connective Tissue

**DOI:** 10.3390/molecules31091505

**Published:** 2026-05-01

**Authors:** Leydy Tatiana Figueroa-Ariza, Willy Cely-Veloza, Miguelángel Coccaro, Diego Fernando Gualtero, Ronald Andrés Jiménez, Ericsson Coy-Barrera, Ana Delia Pinzón-García, Yamil Lesmes, Leandro Chambrone, Gloria Inés Lafaurie

**Affiliations:** 1 Unidad de Investigación Básica Oral—UIBO, Vicerrectoría de Investigación, Facultad de Odontología, Universidad El Bosque, Bogotá 111321, Colombia; lfigueroaa@unbosque.edu.co (L.T.F.-A.); wcelyv@unbosque.edu.co (W.C.-V.); gualterodiego@unbosque.edu.co (D.F.G.); adpinzon@unbosque.edu.co (A.D.P.-G.); chambrone@hotmail.com (L.C.); 2 Bioorganic Chemistry Laboratory—InQuiBio, Facultad de Ciencias Básicas y Aplicadas, Universidad Militar Nueva Granada, Cajicá 250247, Colombia; ericsson.coy@unimilitar.edu.co; 3 Maestría en Ciencias Odontológicas, Facultad de Odontología, Universidad El Bosque, Bogotá 111321, Colombia; mcoccaro@unbosque.edu.co; 4 Grupo de Investigación en Química Aplicada—INQA, Facultad de Ciencias, Universidad El Bosque, Bogotá 111321, Colombia; rajimenez@unbosque.edu.co; 5 Zimplant & Compañía SAS, Bogotá 110121, Colombia; yamil.lesmes@zimplant.com

**Keywords:** electrospinning, *Triticum vulgare* extract, polycaprolactone, periodontal regeneration, fibroblasts, wound healing, drug release kinetics

## Abstract

Electrospun poly(ε-caprolactone) (PCL) membranes incorporating *Triticum vulgare* extract (TVE) were developed as biomimetic scaffolds for periodontal regeneration. Using a ternary solvent system, two experimental formulations (µF-P10 and µF-P10T1) were fabricated and compared against a commercial dermal matrix. SEM analysis revealed bimodal fiber distributions (0.77–1.74 µm) and a surface porosity of 29.86% for TVE-loaded membranes, significantly higher than that of the commercial control (25.26%). FT-IR confirmed that the PCL chemical integrity was preserved, while mechanical testing showed that extract incorporation reinforced the matrix, increasing the Young’s modulus from 2.90 × 10^3^ Pa to 3.54 × 10^3^ Pa. UHPLC–MS identified ferulic acid as the primary bioactive component (90%), with release kinetics following a first-order model (R^2^ = 0.998) over 48 h. Biological assays with human gingival fibroblasts (HGF) confirmed non-cytotoxicity (>70% viability). While both membranes supported healing, the µF-P10 formulation showed superior performance, with 80.2% proliferation and 60.6% wound closure, approaching control levels. These findings demonstrate that PCL-TVE electrospun scaffolds effectively combine favorable morphology and controlled release, offering a promising alternative for subepithelial connective tissue regeneration.

## 1. Introduction

Gingival recession is a condition that affects about 66% of people worldwide. It happens when the gum line moves down, exposing the roots of the teeth [1]. This exposure can lead to concerns about appearance, tooth sensitivity, and a higher risk of root cavities [2,3,4]. There are several reasons why gingival recession occurs, including mechanical trauma, chronic gum inflammation, unusual muscle attachments, and a thin periodontal ligament [4,5]. The likelihood of experiencing this issue increases significantly with age and the presence of various risk factors, with some global estimates suggesting that over 78% of certain populations are affected [1,6]. When it comes to treatment, the goal is to achieve controlled root coverage, increase the thickness of keratinized tissue, and enhance patient comfort from both functional and esthetic viewpoints.

The subepithelial connective tissue graft (CTG) is considered the gold standard for achieving root coverage because of its reliable outcomes, high success rates, and long-lasting results [4,7]. However, this method has notable drawbacks, including donor-site discomfort, limited availability of grafts, and postoperative pain [4,7,8]. To avoid the need for a second surgical site, Acellular Dermal Matrix (ADM) has been introduced as an alternative graft material. ADMs help with root coverage by promoting cell migration from the surrounding gum tissues [9], but they can be quite expensive, have inconsistent integration with the tissue, and have limited natural bioactivity [10]. These challenges have sparked interest in creating synthetic, biofunctional substitutes that offer good mechanical properties and an extracellular matrix (ECM)-like structure, thereby positively influencing cellular and tissue responses [11].

In this context, electrospinning has become one of the most adaptable methods for creating micro- and nanofibrous scaffolds that closely resemble the structure and organization of the natural extracellular matrix (ECM) [12,13,14]. By adjusting the process parameters and the type of polymer used, we can produce fibrous networks with specific shapes and impressive surface-area-to-volume ratios, ranging from simple one-dimensional forms to complex, highly porous three-dimensional structures [15]. These design features enhance cell adhesion, growth, and differentiation, making electrospun membranes exciting options for tissue engineering [16].

In the field of periodontology, these biomimetic platforms can replicate the native ECM, support the migration of progenitor cells such as fibroblasts and periodontal ligament stem cells, and help manage local inflammation [17]. Electrospun fibers have already shown promise in areas like skin regeneration [18], peripheral nerve repair [19], and periodontal treatments, thanks to their high porosity, adjustable mechanical properties, and ability to incorporate biomolecules, nanoparticles, or drugs in either uniform or strategically placed ways [20,21]. This capability allows for the creation of controlled-release systems that can fine-tune the healing environment over time [22].

Among synthetic polymers used in electrospinning, polycaprolactone (PCL) stands out as a popular choice. This FDA-approved aliphatic polyester is well regarded for its biocompatibility, solid mechanical stability, and straightforward processing [23,24]. The electrospun PCL fibers are known for their impressive tensile strength and ductility, reaching up to 1.5 MPa [25], and they have controllable degradation rates that help maintain structural support during crucial regeneration phases [26]. However, PCL does have its downsides: its natural hydrophobicity can hinder surface wettability and affect how cells interact with the material, and it lacks inherent bioactivity [27].

Human gingival mesenchymal stromal cells (hG-MSCs) were cultured in a Gelatin/Polycaprolactone prototype (GPP) or in a collagen matrix (VSCM), showing an enhanced response in GPP, with increased gene expression of growth factors such as VEGF-A, TGF-β1, and α-SMA that occurred until 3 days, demonstrating the potential of electrospun nanofibers as substitutes for soft tissue regeneration [28]. Recently, a bovine-derived, three-layered structure with two external gelatin layers and a central electrospun PCL layer (AGM) was evaluated and compared with a free gingival graft (FGG) for increasing the width of keratinised tissue (KT) in a randomized clinical trial in humans. Although the short-term results with the AGM were promising, they were not sustained after 3 years [29]. To address these challenges associated with its hydrophobic structure and its limited clinical response, researchers have investigated various strategies, such as modifying the surface, blending with more hydrophilic polymers, or adding bioactive compounds to enhance biological performance [30,31].

One particularly exciting method involves adding plant extracts that are rich in antioxidants, anti-inflammatory agents, and compounds that promote regeneration [32]. Numerous studies have shown that the sustained release of these phytochemicals from polymeric matrices can significantly enhance cell proliferation, angiogenesis, and mesenchymal stem cell differentiation [33,34]. For example, Hosseinzadeh et al. (2017) created electrospun nylon 66 scaffolds infused with beetroot (*Beta vulgaris*) extract and tested them in a co-culture system with human keratinocytes and mesenchymal stem cells [21]. They found a notable increase in cell proliferation and an upregulation of epithelial markers (cytokeratins 10 and 14) and dermal proteins [21]. In a similar vein, phenolic compounds such as veratric acid, when encapsulated in chitosan nanoparticles within coaxial PCL/PVP fibers, have demonstrated sustained release profiles and improved osteoblastic differentiation in murine mesenchymal stem cells [20].

Additionally, lignin—a plant-derived antioxidant polymer—when incorporated into PCL fibers, has been shown to enhance cell viability and promote neuronal differentiation both in vitro and in vivo [16]. Comparative studies suggest that combining PCL with bioactive compounds can yield even better results [18].

*Triticum vulgare*, commonly known as wheat, stands out as a phytocompound that has garnered significant attention. Its extracts are rich in bioactive molecules, including ferulic acid, tocopherols (which include vitamin E), and other polyphenols, all of which are well known for their antioxidant and anti-inflammatory benefits [35,36,37,38]. When applied topically, *T. vulgare* extracts have been shown to promote soft tissue regeneration by stimulating fibroblast growth and collagen production [39,40]. Additionally, its antioxidant properties help reduce oxidative stress in damaged areas, while its anti-inflammatory elements help steer the local immune response towards a more regenerative state [37]. By encapsulating *T. vulgare* in electrospun PCL fibers, we can potentially merge the mechanical strength and biomimetic structure of the polymer with the extract’s biological benefits, creating a scaffold that supports the regeneration of subepithelial connective tissue.

The current study is set to create and analyze nanofibrous PCL membranes, both with and without the addition of *T. vulgare* extract. We will compare their morphological, mechanical, and functional characteristics with those of a commercially available acellular dermal matrix. To ensure these membranes are suitable substitutes for subepithelial connective tissue, we will evaluate their biocompatibility and regenerative potential through cell-based assays as an initial step towards preclinical validation.

## 2. Results

### 2.1. Rheological Characterization of Spinning Solutions

The rheological analysis revealed that the incorporation of *Triticum vulgare* extract (TVE) significantly modifies the viscosity profile of the PCL solution. The neat PCL solution (μF-P10) exhibited a higher zero-shear viscosity of 1750 mPa·s (Table 1), which provides a high degree of polymer chain entanglement, favoring the formation of uniform, cylindrical fibers observed in SEM.

In contrast, the addition of TVE (μF-P10T1) resulted in a reduction in the zero-shear viscosity to approximately 1200 mPa·s (Table 1). This viscosity drop suggests that the bioactive components of the extract—likely small phenolic molecules such as ferulic acid and polysaccharides—interfere with PCL-PCL intermolecular interactions, acting as plasticizing agents.

This decrease in viscosity profile (Figures S1 and S2 in Supporting Information), coupled with the expected increase in electrical conductivity from the extract’s polar compounds, explains the transition toward a bimodal fiber distribution. A lower viscosity allows the electric field to stretch the jet more extensively, leading to the formation of thinner nanofibers, while rapid solvent evaporation in the ternary system induces the collapse of the fiber shell into a ribbon-like geometry. These findings directly address the reviewer’s concerns regarding process stability, confirming that the observed morphologies are a controlled physical consequence of the solution’s rheological properties.

### 2.2. Morphological Characterization of Membranes

**Figure 1:**
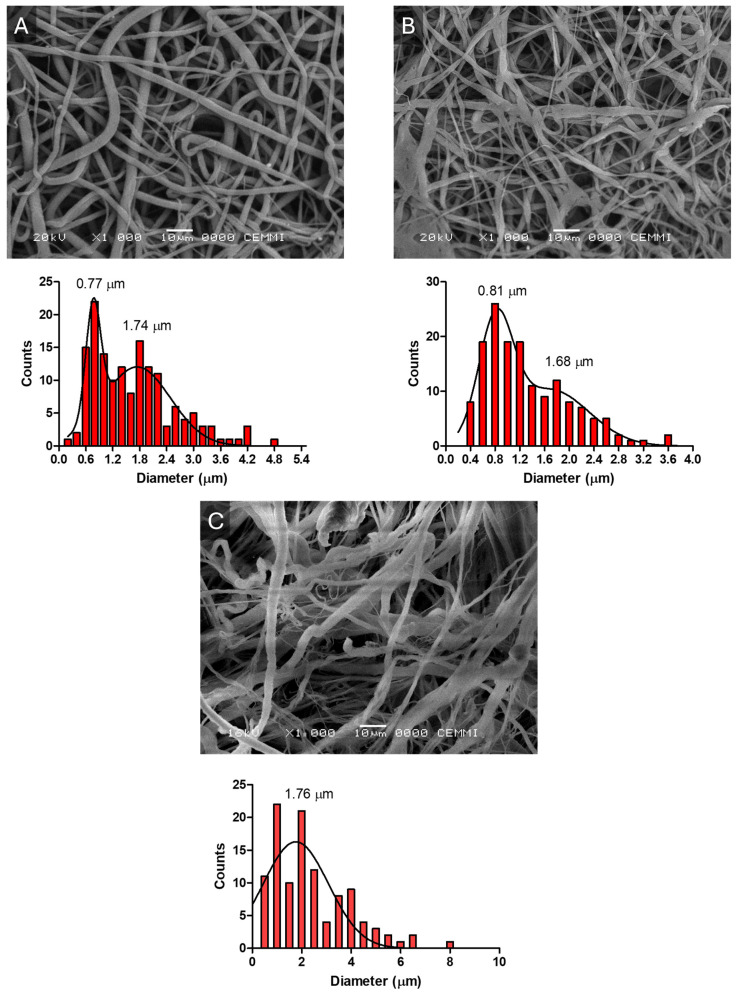
Representative SEM micrographs and fiber diameter distribution histograms of the electrospun membranes and the commercial dermal matrix. (**A**) The µF-P10 membrane showed a bimodal distribution with peaks at 0.77 µm and 1.74 µm. (**B**) The µF-P10T1 membrane displayed a bimodal distribution with fiber diameters of 0.81 µm and 1.68 µm, whereas (**C**) the commercial dermal matrix (Dermis) exhibited a unimodal distribution with an average fiber diameter of 1.76 µm. Scale bars are indicated in each image. Histograms were generated from measurements taken in 200 randomly selected regions per sample using ImageJ 1.54p (National Institutes of Health, Bethesda, MD, USA).

The morphology of the electrospun membranes was examined using scanning electron microscopy (SEM) to assess fiber diameter distribution, surface porosity, and structural integrity. Representative SEM micrographs and corresponding fiber diameter histograms are shown in Figure 1. The µF-P10 membrane (Figure 1A) showed a bimodal distribution with peaks at 0.77 µm and 1.74 µm, and µF-P10T1 (Figure 1B) also exhibited a bimodal distribution with fiber diameters of 0.81 µm and 1.68 µm. The commercial dermal matrix (Dermis) (Figure 1C) demonstrated a unimodal distribution with an average fiber diameter of 1.76 µm.

**Figure 2:**
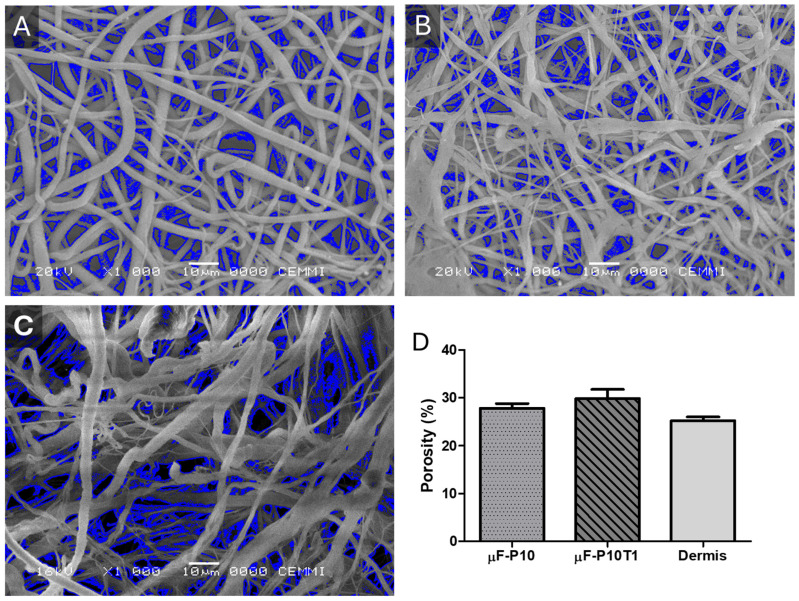
Surface porosity of electrospun membranes and the commercial dermal matrix (**A**) µF-P10, (**B**) µF-P10T1, (**C**) Dermis, and (**D**) Percentage of Porosity. Porosity was quantified from SEM images by calculating the percentage of open surface area relative to the total field of view. Bars represent the mean ± SD (*n* = 3) for each membrane type. The µF-P10 and Dermis exhibited comparable porosity levels, whereas µF-P10T1 presented slightly higher surface porosity.

Surface porosity analysis (Figure 2D) revealed mean porosity values of 27.81 ± 1.70% for µF-P10, 29.86 ± 3.30% for µF-P10T1, and 25.26 ± 1.24% for Dermis. These results indicate that, although µF-P10 (Figure 2A) and Dermis (Figure 2C) shared similar fiber diameters at one of their peaks, µF-P10T1 (Figure 2B) featured slightly thinner fibers and a somewhat higher surface porosity.

To statistically validate the observed bimodality, fiber diameter measurements were taken in 200 randomly selected regions for each sample, using 3 independent SEM micrographs analyzed with ImageJ 1.54p software (National Institutes of Health, Bethesda, MD, USA).

The volumetric porosity measurements revealed a reduction in the void space upon the incorporation of the phytotherapeutic extract. The neat PCL membranes (μF-P10) exhibited a porosity of 53.26%, whereas the loaded membranes (μF-P10T1) showed a porosity of 42.68%. This trend indicates that the presence of *Triticum vulgare* extract and the resulting changes in fiber morphology contribute to a more densely packed fibrous network.

### 2.3. Mechanical Properties of Membranes

**Figure 3:**
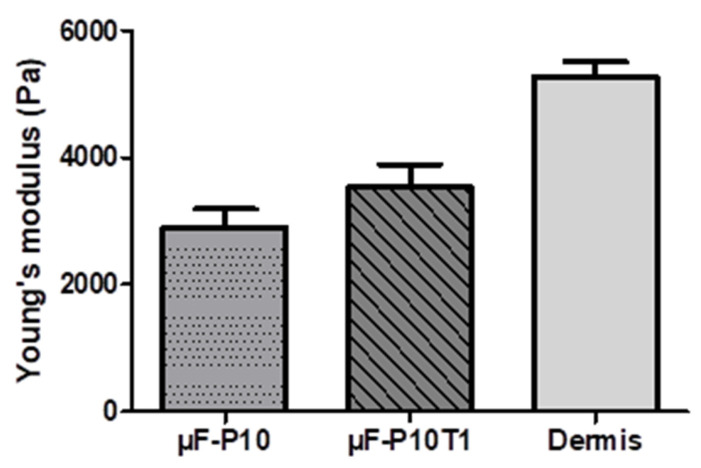
Young’s modulus of electrospun membranes and acellular dermis (µF-P10 membrane, µF-P10T1 membrane, and commercial dermal matrix (Dermis)).

Young’s modulus was determined using uniaxial tensile tests. As shown in Figure 3, the unloaded membrane (µF-P10) had an average modulus of 2900.47 ± 500.19 Pa, while the membrane loaded with plant extract (µF-P10T1) increased to 3540.06 ± 607.68 Pa. The acellular dermis, on the other hand, exhibited the highest value at 5276.39 ± 408.66 Pa. These results demonstrate a progressive increase in the material’s stiffness with the load of the plant extract. The membrane loaded with the plant extract was approximately 1.22 times stiffer than the unloaded membrane; however, it was not as stiff as the human acellular dermis, which was 1.5 times stiffer than the membrane loaded with the extract.

### 2.4. Physicochemical Properties of Membranes

**Figure 4:**
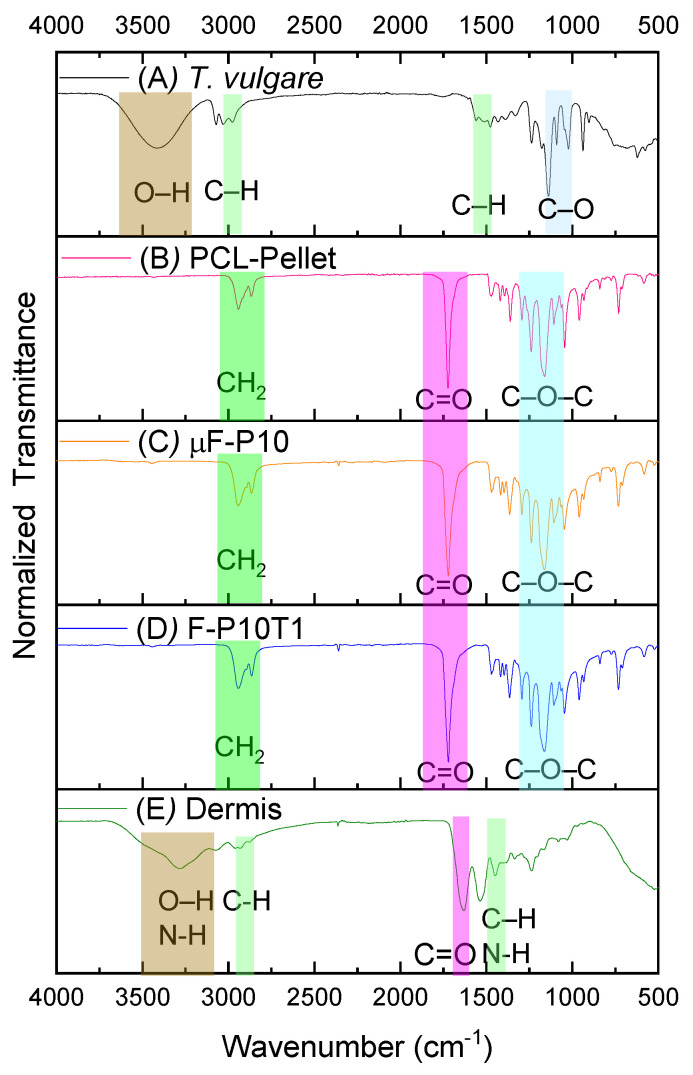
Normalized FT-IR spectra of the reference materials and electrospun membranes: (**A**) *T. vulgare* extract (black line), (**B**) PCL–pellet (pink line), (**C**) µF-P10 membrane (orange line), (**D**) µF-P10T1 membrane (blue line), and (**E**) commercial dermal matrix (Dermis, green line). The colored shaded regions highlight characteristic functional groups: brown for O–H and N–H stretching; light green for C–H and C–N; magenta for C=O stretching; and cyan for C–O and C–O–C stretching.

The chemical composition of the electrospun membranes and reference materials was analyzed using Fourier-transform infrared (FT-IR) spectroscopy. Representative spectra are presented in Figure 4, in the following order: *T. vulgare* extract (Figure 4A), PCL–pellet (Figure 4B), µF-P10 (Figure 4C), µF-P10T1 (Figure 4D), and Dermis (Figure 4E).

### 2.5. Quantification of Bioactive Component Delivery from Membrane

**Figure 5:**
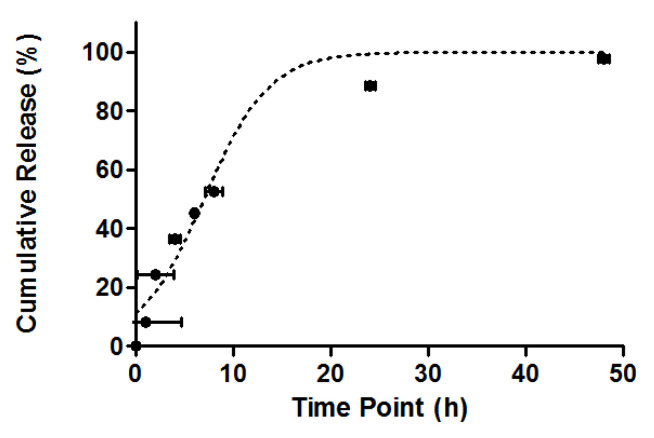
*T. vulgare*-loaded membranes release in PBS (pH 7.4), showing an initial moderate release of 60% after 10 h and a complete extract release after 48 h. Release data are presented as normalized cumulative release (%) to facilitate comparison among specimens and to minimize the effect of minor differences in membrane mass and initial extract loading.

The *T. vulgare*-loaded fibers release study showed an initial moderate release of approximately 60% after 10 h, followed by an 88% extract release at 24 h, and finally, complete release after 48 h. This moderate release rate of *T. vulgare* is attributed to the hydrophobic nature of the PCL polymer, which slows the disintegration and dissolution of the membranes. The presence of 80% propylene glycol (hydrophilic) in the commercial extract of *T. vulgare* may also be a cause of the slow release kinetics, considering the hydrophobic nature of the PCL membranes included in this investigation. Finally, it is important to mention that, despite the slow release of the extract as a function of time, the behavior of the release rate agrees with the work reported by Essam A. Tawfik et al. (2022) [41], as can be seen in Figure 5.

**Figure 6:**
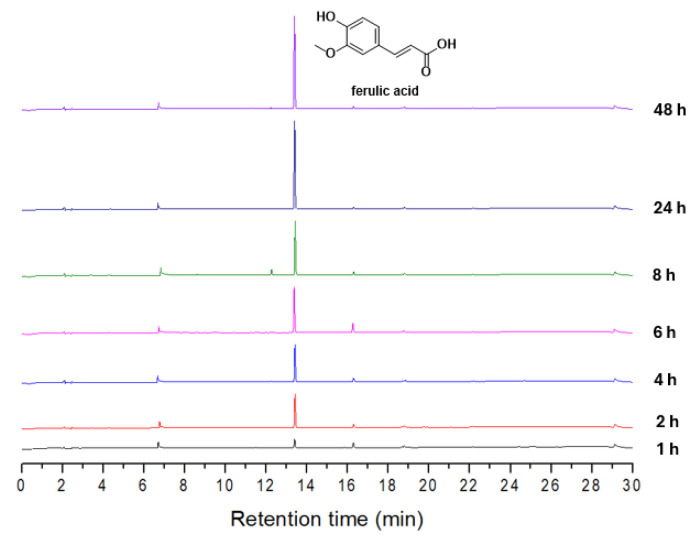
Representative LC-MS chromatogram of the *T. vulgare* extract. The primary compound detected corresponds to ferulic acid, with a retention time (Tr) of 13.5 min and a relative abundance of approximately 90%.

**Figure 7:**
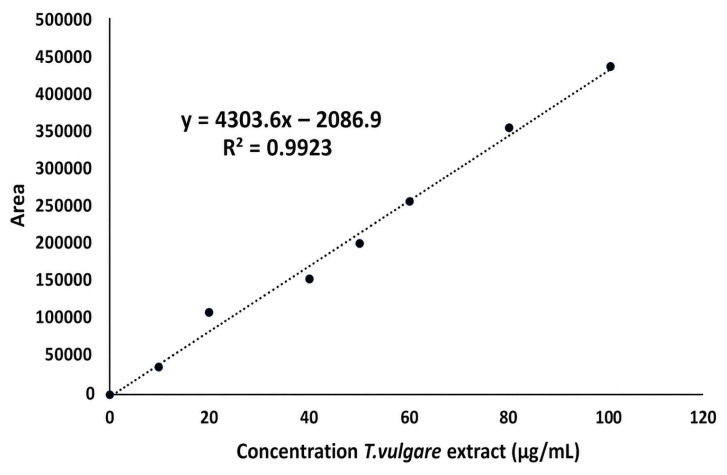
Calibration curve of *T. vulgare* extract in the concentration range of 10–100 ppm. The linear regression equation obtained was y = 4303.6x − 2086.9, with a correlation coefficient R^2^ = 0.9923.

The quantification of the release of *T. vulgare* extract was performed using the primary component detected by liquid chromatography coupled to mass spectrometry (LC-MS). The major component of the extract corresponds to ferulic acid (Tr 13.5 min) (Figure 6), which is present at a relative abundance of 90%. Using the calibration curve of *T. vulgare* at concentrations between 10 and 100 ppm (Figure 7), the equation of the line y = 4303.6x − 2086.9 (R^2^ = 0.9923) was obtained, with which the areas obtained below were interpolated. The corresponding concentrations of the released extract were calculated as a function of time, as shown in the kinetic graph in Figure 6.

**Figure 8:**
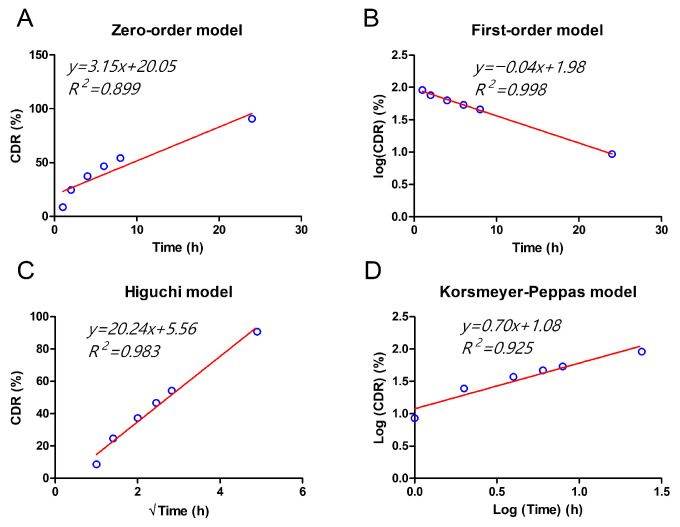
Fitting of the release profile of *T. vulgare*-loaded membranes to different kinetic models: (**A**) Zero-order, (**B**) first-order, (**C**) Higuchi, and (**D**) Korsmeyer–Peppas. Cumulative Drug Release (% CDR) is plotted as a function of time.

To better understand the release mechanism of the *T. vulgare* extract from the electrospun membranes, the experimental data were fitted to various kinetic models commonly used in drug release studies, including zero-order, first-order, Higuchi, and Korsmeyer–Peppas models. The correlation coefficients (R^2^) obtained for each model are presented in Figure 8, allowing identification of the most suitable model to describe the release profile [42].

Among the evaluated kinetic models, the zero-order model showed the lowest correlation with the experimental data (Figure 8A, R^2^ = 0.899), indicating that a constant release rate over time does not adequately describe the system. The Korsmeyer–Peppas model provided a slightly better fit (Figure 8D, R^2^ = 0.925), suggesting limited applicability to the release mechanism under the current conditions. In contrast, the Higuchi model exhibited a strong linear correlation (Figure 8C, R^2^ = 0.983), implying that diffusion plays a relevant role in the release process. Notably, the first-order model showed the best fit to the experimental data (Figure 8B, R^2^ = 0.998), indicating that the release of *T. vulgare* is predominantly concentration-dependent.

### 2.6. Stimulation of FGH with Membranes Loaded with T. vulgare

#### 2.6.1. Cell Viability Assay

Cell viability was evaluated using the alamarBlue assay, with values expressed as a percentage relative to untreated control wells (set at 100%). After 24 h of exposure, the control group showed a viability of 100.00 ± 2.30%. Cells treated with µF-P10 exhibited a reduced viability of 79.94 ± 10.03%, whereas µF-P10T1 maintained 89.73 ± 12.16%.

**Figure 9:**
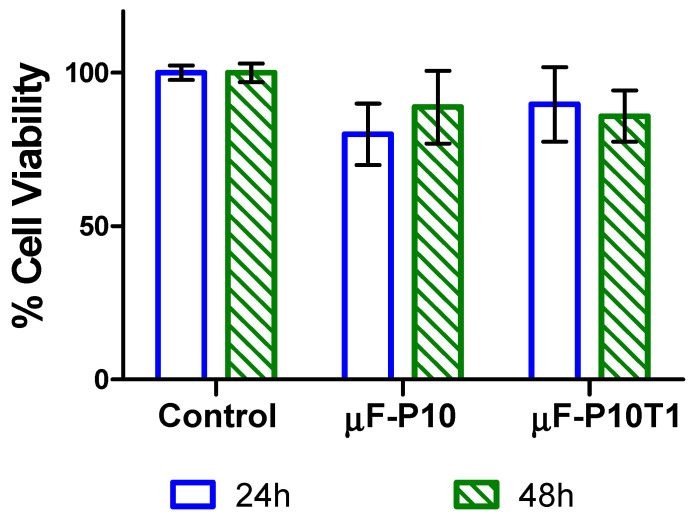
Cell viability of FGH cultures after exposure to experimental membranes. Viability was assessed using the Alamar Blue assay after 24 h and 48 h of stimulation with µF-P10 and µF-P10T1 membranes. Data are expressed as the percentage of viable cells relative to the untreated control (set at 100%). Bars represent the mean ± standard deviation (SD) from six independent replicates per condition. None of the treatments reduced viability to below 70% at any time point, indicating the absence of significant cytotoxicity.

At 48 h, the viability of the control group was 100.00 ± 3.02%, while µF-P10 and µF-P10T1 showed viabilities of 88.83 ± 11.86% and 85.87 ± 8.37%, respectively. In both exposure periods, none of the treatments reduced viability to below 70%, indicating no significant cytotoxicity under the tested conditions (Figure 9).

#### 2.6.2. Cell Proliferation Assay

Cell proliferation in FGH cultures was assessed after 48 h of stimulation with µF-P10, µF-P10T1, or a commercial dermal matrix (Dermis), using the Cell-Quant™ No Wash Cell Proliferation Assay. Proliferation values were expressed as a percentage relative to the untreated control (set at 100%). The control group showed 100.00 ± 13.76% proliferation. µF-P10-treated cultures showed 80.21 ± 24.26%, while µF-P10T1 exhibited markedly lower proliferation at 48.98 ± 16.38%. Dermis-treated cultures also exhibited reduced proliferation (48.35 ± 22.80%).

**Figure 10:**
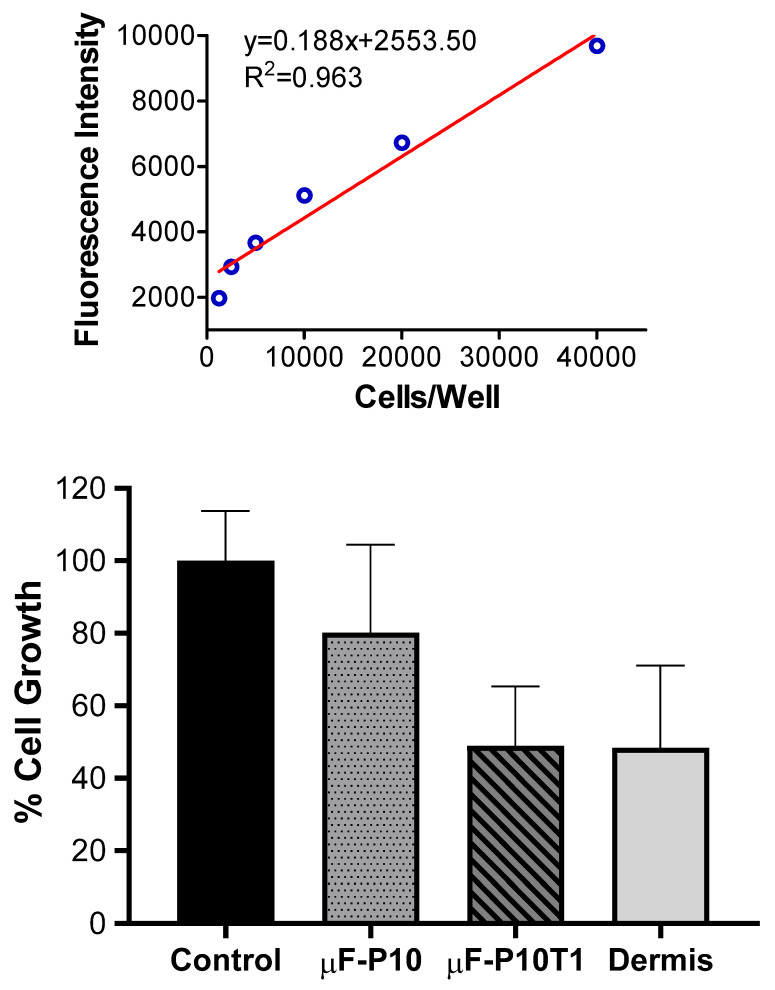
*Cell proliferation of FGH cultures after 48 h of stimulation with experimental membranes or a commercial dermal matrix.* Proliferation was determined using the Cell-Quant™ No Wash Cell Proliferation Assay and expressed as a percentage relative to the untreated control (set at 100%). Bars represent the mean ± SD (n = 6 for Control, µF-P10, and µF-P10T1; n = 4 for Dermis). µF-P10 supported higher proliferation rates compared with µF-P10T1 and Dermis, both of which significantly reduced cell growth.

Overall, µF-P10 supported proliferation at levels closer to the control, whereas µF-P10T1 and Dermis significantly limited cell growth under the tested conditions (Figure 10).

#### 2.6.3. Evaluation of Wound Closure in Response to Membrane Stimulation

**Figure 11:**
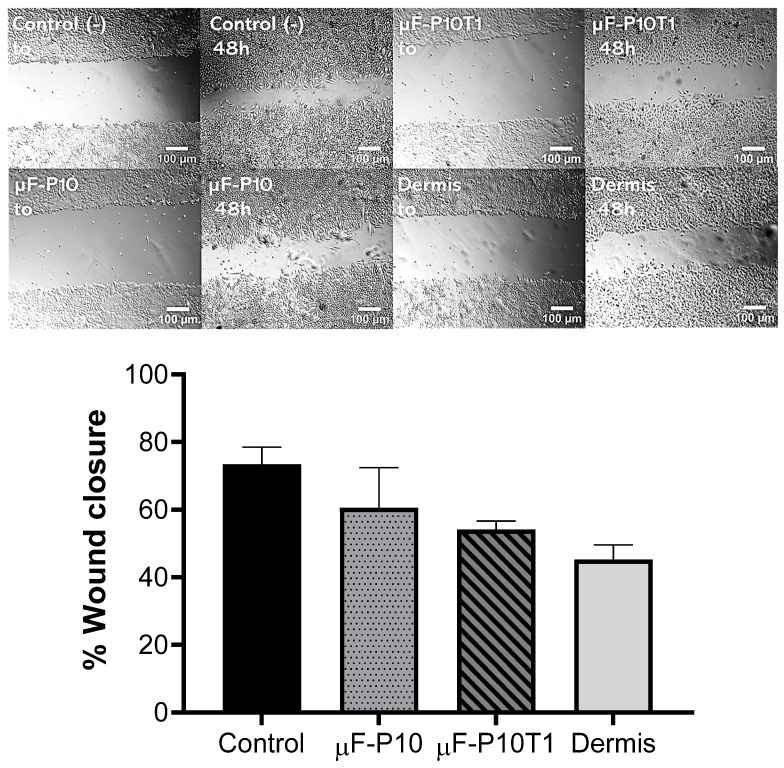
*Wound closure in FGH monolayers after 48 h of stimulation with experimental membranes or a commercial dermal matrix.* Representative phase-contrast micrographs (20× magnification) at baseline (*t*_0_) and after 48 h (*t*_48_*h*) are shown for each treatment group: untreated control, µF-P10, µF-P10T1, and commercial Dermis (Puros^®^ Dermis Allograft Tissue Matrix). The same wound site was imaged at both times for consistency. The lower panel displays the quantitative analysis of wound closure percentage, calculated from the reduction in wound area between *t*_0_ and *t*_48_*h* using ImageJ 1.54p software (National Institutes of Health, Bethesda, MD, USA). Bars represent the mean ± SD (n = 3). The control and µF-P10 groups achieved higher closure percentages compared with µF-P10T1 and Dermis.

Wound healing capacity was assessed in FGH monolayers following scratch induction and 48 h of treatment with the experimental membranes or a commercial dermal matrix. Representative images of wound areas at baseline (*t*_0_) and after 48 h are shown in Figure 11.

Quantitative analysis revealed that the untreated control group achieved the highest wound closure rate, with 73.38 ± 5.18% closure after 48 h. µF-P10-treated cultures exhibited a comparable closure rate of 60.57 ± 11.92%, whereas µF-P10T1 achieved 54.05 ± 2.67% closure. The Dermis group showed the lowest closure rate, at 45.23 ± 4.37%.

Overall, µF-P10 maintained wound closure levels closer to the control, while µF-P10T1 and Dermis demonstrated reduced closure efficiency under the tested conditions.

## 3. Discussion

The present study demonstrates that electrospun PCL membranes loaded with *T. vulgare* extract exhibit morphological, physicochemical, and biological properties that support their potential as scaffolds for subepithelial connective tissue regeneration.

### 3.1. Rheological Characterization of Spinning Solutions

The stability of the electrospinning process and the resulting fiber architecture were deeply influenced by the rheological properties of the spinning solutions (see Figures S1 and S2 in the Supplementary Material). Both μF-P10 and μF-P10T1 exhibited a pronounced shear-thinning behavior, which is essential for maintaining a stable Taylor cone while allowing the jet to thin efficiently under the high shear stress of the electric field.

The significant drop in zero-shear viscosity observed upon the addition of *Triticum vulgare* extract, from 1750 mPa·s to 1200 mPa·s, suggests that the bioactive compounds of TVE (e.g., ferulic acid and oligosaccharides) act as molecular spacers. This interaction reduces the intermolecular friction between PCL chains, increasing the free volume of the solution.

This rheological shift directly explains the transition from the uniform cylindrical fibers of μF-P10 to the bimodal and ribbon-like morphology of μF-P10T1. The lower viscosity in the loaded solution, combined with the rapid solvent evaporation of the ternary system, facilitates the collapse of the fiber shell during flight—a physical phenomenon consistent with high Peclet numbers.

From a tissue engineering perspective, this “viscosity-driven” bimodal architecture is not a defect but a functional advantage. The hierarchy of fine and coarse fibers mimics the natural organization of the periodontal extracellular matrix (ECM), providing a high-surface-area template that enhances the attachment of human gingival fibroblasts (HGF) and ensures the sustained release of the encapsulated antioxidant compounds.

### 3.2. Morphology and Porosity

The formation of ribbon-like fibers observed in the electrospun µF-P10 and µF-P10T1 membranes can be rationalized based on solvent evaporation kinetics coupled with electrohydrodynamic jet dynamics. The ternary solvent system (chloroform/dichloromethane/methanol, 60:30:10 *v*/*v*/*v*) combines solvents with different vapor pressures and boiling points, leading to non-uniform solvent evaporation along the jet trajectory.

Chloroform and dichloromethane exhibit high vapor pressures and low boiling points, promoting rapid solvent evaporation at the jet surface. This results in the formation of a solidified polymer shell, while the jet’s core may still contain residual solvent. The evolution of this core–shell structure is governed by the competition between solvent diffusion and evaporation, which can be described by a characteristic Peclet number:

Pe=(R·v)/D

where R is the jet radius, v is the axial velocity, and D is the solvent diffusion coefficient. At high Pe values, solvent evaporation dominates over diffusion, favoring the formation of a glassy outer layer that can collapse under capillary forces. This collapse transforms initially cylindrical fibers into flattened or ribbon-like structures.

Additionally, the incorporation of *Triticum vulgare* extract modifies the physicochemical properties of the spinning solution. The presence of polar components (glycols or phenolic compounds) increases solution viscosity and reduces solvent diffusivity, further enhancing the formation of a solvent-rich core. The elongational viscosity (*ηₑ*) plays a critical role in resisting jet thinning, and its increase can be associated with reduced stretching efficiency according to:

$$d_{f}\propto {\left(\frac{\eta }{\varepsilon_{0}E^{2}}\right)}^{\frac{1}{3}}$$

where $d_{f}$ is the fiber diameter, *η* the viscosity, *ε*_0_ the permittivity of free space, and *E* the electric field. As a result, fibers in µF-P10T1 may reach the collector with a partially solvent-filled core, making them susceptible to mechanical flattening upon impact.

The stability of the encapsulated bioactive compounds is enhanced by the fiber formation dynamics during electrospinning. In the ternary solvent system used, the process is governed by a high Peclet number (Pe > 1), indicating that the rate of solvent evaporation at the jet surface is much faster than the diffusion of the solvent from the core to the exterior. This thermodynamic imbalance causes the near-instantaneous formation of a dense, solid polymeric “skin” or shell that acts as a protective physical barrier. This surface vitrification not only causes the fibers to collapse into a ribbon-like geometry as the residual solvent evaporates from the core, but also effectively protects the T. vulgare extract from environmental factors such as oxygen and light, reducing the risk of premature oxidation and preserving its bioactivity until the release phase.

SEM analysis revealed bimodal fiber distributions for µF-P10 and µF-P10T1, with diameters ranging from 0.7 to 1.7 µm, whereas the commercial dermal matrix exhibited a unimodal distribution (1.76 µm). These values are within the range reported for nanofibrous scaffolds that promote periodontal tissue regeneration, where fiber sizes below 2 µm enhance cell adhesion and extracellular matrix deposition [11,22]. µF-P10T1 displayed slightly thinner fibers and the highest porosity (29.9%), which could favor nutrient diffusion and cell infiltration—a critical feature for tissue integration [19]. This bimodality can be explained by fluctuations in jet stability under conditions near the polymer solution’s critical entanglement concentration. In such regimes, small variations in local viscosity, charge density, and solvent evaporation rate can induce different elongational pathways within the whipping jet, leading to the simultaneous formation of nano- and micro-scale fibers.

The observed decrease in volumetric porosity (from 53.26% to 42.68%) in the μF-P10T1 scaffolds is consistent with the morphological transition toward finer, ribbon-like fibers. The lower viscosity of the loaded solution allows for greater jet stretching, resulting in a more compact fiber arrangement. While the total void volume is reduced, the effective surface area for human gingival fibroblast (HGF) interaction is enhanced. Furthermore, a porosity of 40–50% provides an optimal balance between structural integrity for surgical handling and the necessary interconnectivity for cellular infiltration and metabolic exchange in the periodontal environment.

### 3.3. Mechanical Properties of Membranes

The loading of the plant extract significantly increased the Young’s modulus of the membrane, suggesting a structural reinforcement effect attributable to interactions between the extract’s phenolic compounds and the polymeric membrane, which promote cross-linking and reduce molecular mobility. This behavior is consistent with recent reports where plant extracts act as natural cross-linking agents, improving mechanical strength and stability [34,43]. This behavior is relevant in the dental field, where polymeric and dermal matrices are used as soft tissue substitutes in periodontal procedures, mucogingival regeneration, and root coverage. An intermediate stiffness, such as that provided by the plant extract, could offer an adequate balance among mechanical support, tissue integration, and conformability, favoring functional and esthetic regeneration in oral and maxillofacial applications.

### 3.4. Physicochemical Properties

FTIR spectra confirmed the preservation of PCL’s chemical structure after electrospinning, with no distinct bands attributable to *T. vulgare* extract. This suggests either low extract loading or signal overlapping below the detection threshold, a finding consistent with previous studies embedding plant extracts in polymeric scaffolds [32,34]. The absence of structural alterations implies that the membranes retain the mechanical and degradation features typical of PCL, which are essential for periodontal applications [23].

The FT-IR spectrum of the *Triticum vulgare* extract showed absorption bands consistent with the presence of ferulic acid as a major phenolic constituent. The broad band around 3400 cm^−1^ was assigned to O–H stretching vibrations from phenolic and carboxylic hydroxyl groups [44]. The signal around 3000 cm^−1^ can be associated with aromatic C–H stretching [44,45]. The band in the 1500 cm^−1^ region is more appropriately related to aromatic C=C stretching vibrations of the ferulic acid structure rather than to generic C–H bending [44,46]. In addition, the absorption region around 1275–1200 cm^−1^, extending toward 1100 cm^−1^, is consistent with C–O and C–O–C stretching vibrations associated with the methoxy and carboxylic functionalities of ferulic acid [44,45,46,47].

The spectrum of the PCL–pellet exhibited the characteristic bands of polycaprolactone: 3000 cm^−1^ for asymmetric and symmetric CH_2_ stretching [48,49], 1750 cm^−1^ for carbonyl (C=O) stretching [50,51], 1290–1293 cm^−1^ for C–O and C–C stretching, and 1200 cm^−1^ for C–O–C stretching [49,50,51]. Since µF-P10 and µF-P10T1 retained these same PCL bands, it is reasonable to state that electrospinning did not significantly alter the polymer backbone.

Finally, the spectrum of the commercial dermal matrix exhibited the typical collagen/protein bands: A broad band near 3330–3300 cm^−1^ assigned to N–H/O–H stretching (amide I) [52,53]. A band near 2930–2948 cm^−1^ assigned to CH_2_ asymmetric stretching (amide II) [54]; the Amide I band near 1650 cm^−1^ related to C=O stretching of peptide bonds [52,53]; and the Amide II band near 1450 cm^−1^ due to C–N stretching [52,53,55].

### 3.5. Controlled Release of the Extract

The release profile of the *T. vulgare*-loaded membranes exhibited an initial moderate release of 60% at 10 h, reaching 88% at 24 h, and complete release after 48 h. This behavior best fit the first-order kinetic model (R^2^ = 0.998), suggesting that the release of the extract is primarily controlled by the concentration of the substance in the released phase, rather than a constant release rate or a diffusion-controlled process.

The initial moderate release can be explained by the hydrophobic nature of the PCL polymer used in the electrospun membranes. This material, being hydrophobic, slows down the dissolution of the membranes and, consequently, the release of the extract. Additionally, the presence of 80% propylene glycol, a hydrophilic excipient in the commercial *T. vulgare* extract, may also contribute to the slower release kinetics, as its interaction with the hydrophobic PCL matrix limits the rapid diffusion of hydrophilic molecules like ferulic acid.

LC-MS analysis of the extract revealed that the primary component identified was ferulic acid, which accounted for approximately 90% of the relative abundance. This compound was detected with a retention time (Tr) of 13.5 min, and its identity was confirmed by the mass spectrometric data, where a peak corresponding to the expected molecular ion (*m*/*z* 194) was observed.

For the quantification of the released ferulic acid at different time points, the areas under the chromatographic peaks (AUC) were analyzed. A calibration curve, established with ferulic acid concentrations between 10 and 100 ppm, and the linear regression equation (y = 4303.6x − 2086.9, R^2^ = 0.9923), were used to interpolate the AUCs corresponding to the released concentrations at each sampling time. This analysis allowed for the precise determination of ferulic acid concentrations at 10, 24, and 48 h, revealing a gradual and concentrated release over time, with a total of 100% release after 48 h.

Fitting the experimental data to different kinetic models showed that the first-order model best described the observed release profile. This behavior is consistent with previous studies on PCL-based controlled-release systems, where the release of hydrophilic compounds, such as ferulic acid, tends to follow a concentration-dependent kinetic pattern. In these systems, the release is influenced by factors such as the compound’s solubility, the polymer’s properties, and the interaction between the active ingredient and the polymer matrix.

Additionally, the Higuchi model (R^2^ = 0.983) and Korsmeyer–Peppas model (R^2^ = 0.925) provided better fits than the zero-order model (R^2^ = 0.899), which did not adequately describe the observed behavior. This suggests that the release does not follow a constant rate but is governed by a diffusion-controlled process, albeit with some concentration dependence.

Ferulic acid, one of the main phenolic compounds in *T. vulgare*, is well known for its antioxidant and anti-inflammatory properties, which could explain the biological responses observed with the use of the *T. vulgare*-loaded membranes [35,36]. These effects are particularly relevant in therapeutic applications, where controlled release of active compounds is desired to maximize efficacy while minimizing side effects. Given the gradual release of ferulic acid, the potential for sustained biological activity over time is significant, which may improve the safety and efficacy profile in clinical or personal care applications.

### 3.6. Biological Performance: Viability, Proliferation, and Wound Closure

FGH viability remained above 70% under all conditions, confirming the membranes’ biocompatibility. Importantly, proliferation was better supported by µF-P10 (80%) than by µF-P10T1 and Dermis, both of which significantly reduced cell growth. Since fibroblast proliferation is crucial for connective tissue regeneration and root coverage procedures [5,56], this finding underscores µF-P10′s biological advantage.

The wound-healing assay further confirmed this trend: µF-P10 achieved closure rates closer to the control (60.6% vs. 73.4%), whereas µF-P10T1 and Dermis exhibited impaired closure (<55%). This outcome may reflect a more balanced release of the extract in µF-P10, maintaining bioactive concentrations without negatively impacting fibroblast proliferation. Previous studies have demonstrated that *T. vulgare* extract accelerates fibroblast migration and tissue repair through antioxidant and immunomodulatory mechanisms [39,40].

Initial fibroblast adhesion on electrospun scaffolds is governed by fiber diameter/topography, surface chemistry (wettability), and porosity, which together modulate focal adhesion formation and downstream integrin–FAK signaling. In our study, both µF-P10 and µF-P10T1 exhibited submicron-to-micron fiber diameters (0.7–1.7 µm) with relatively high surface porosity (≈28–30%), architectural features that enlarge the available surface area for focal adhesion assembly and facilitate cell–material contact. These parameters are consistent with fiber size ranges that support adhesion of periodontal tissue cells and ECM deposition. Mechanistically, integrin engagement on fibrous substrates activates focal adhesion kinase (FAK) to coordinate cytoskeletal anchorage and motility programs—an axis repeatedly implicated in fibroblast spreading and migration. Furthermore, surface wettability of PCL influences nascent adhesion: original studies show that tuning solvent systems or using gentle physicochemical modifications can alter contact angles and improve early fibroblast attachment on PCL nanofibers; collagen coating similarly enhances adhesivity [57,58].

After adhesion, fibroblast proliferation depends on both the scaffold’s biophysical cues (stiffness, fiber architecture) and the local concentration/time profile of bioactives. In our data, µF-P10 supported higher fibroblast proliferation (≈80% of control) compared with µF-P10T1 and Dermis, indicating that the unloaded PCL architecture provided a more permissive proliferative niche under our conditions. The differential effect aligns with two observations: (i) mechanosensing—changes in substrate stiffness modulate fibroblast growth; our membranes showed a step-up in Young’s modulus with extract loading (µF-P10T1 > µF-P10), and independent work demonstrates that fibroblast collective migration/proliferation are stiffness-responsive, with stiffer environments altering directionality and speed; (ii) bioactive dose/kinetics—UHPLC-MS identified ferulic acid (≈90% relative abundance) as the dominant component released from µF-P10T1, with complete release by 48 h; although phenolic phytochemicals can enhance fibroblast proliferation and wound repair, dose and timing are critical for a net pro-regenerative effect. Together, these data suggest that moderate mechanical cues with controlled, non-excessive polyphenol exposure (as in µF-P10 under our in vitro settings) favored cell cycle progression, whereas higher early local concentrations and/or altered stiffness in µF-P10T1 may have tempered proliferation [59,60].

Fibroblast-mediated wound closure and ECM remodeling require efficient migration along and through the fibrous network, protease activity, and new collagen deposition. In our scratch assay, µF-P10 achieved wound closure closer to the untreated control than µF-P10T1 or Dermis, indicating that the base PCL architecture supported more effective migratory dynamics. Contact guidance from fibrous topographies is known to bias fibroblast morphology and migration trajectories, with aligned or ordered architectures increasing directional motility—an effect demonstrated in original studies using engineered alignment and complex fiber crossings. Beyond guidance, porosity and pore size critically determine whether cells infiltrate or remain superficial; recent quantitative imaging in electrospun PCL shows that modest porosity shifts produce large pore-size changes that directly impact cell ingress, and that, in vivo, increasing pore area enhances host tissue penetration and neovascularization of PCL nanofibers. Finally, fibroblasts on PCL/collagen hybrids deposit ECM with altered composition and improved handleability—evidence that scaffold chemistry and architecture jointly influence collagen/fibronectin deposition and the balance of matrix remodeling [61,62].

Integrating these mechanisms with our results, we propose that µF-P10 provides a balanced interplay of (i) fiber diameters in the sub-micron to micron range and (ii) surface porosity that supports focal adhesion formation and migration without impeding cell transit; this results in higher proliferation and faster wound closure relative to µF-P10T1 and Dermis. In µF-P10T1, despite a modest increase in stiffness and broadly similar fiber diameters/porosity, the release of ferulic-acid-rich extract—while beneficial in many wound models—may have produced an early local concentration and mechanical context that dampened proliferation and migration under our assay conditions; optimizing extract loading or layered architectures that decouple mechanical reinforcement from immediate pericellular dosing could further enhance outcomes [63].

### 3.7. Perspective and Limitations

The novelty of this study lies in the incorporation of Triticum vulgare extract into electrospun fibers, specifically designed as a scaffold for the regeneration of subepithelial connective tissue. The commercial Triticum vulgare extract was selected based on its well-documented phytotherapeutic properties, including its ability to stimulate fibroblast activity, accelerate wound healing, and promote regeneration of connective tissues [40,64,65,66]. Unlike previous studies that mostly employed aqueous extracts rich in oligosaccharides, this work uses an extract enriched in secondary metabolites, particularly ferulic acid, which has demonstrated cicatrizing, antioxidant, and anti-inflammatory properties [67]. While the efficacy of these compounds in humans has not been extensively evaluated, their potential in regenerative medicine is promising. By combining controlled release of this bioactive extract with the physical structure of electrospun fibers, the study provides a scaffold that supports cellular activity and tissue repair, distinguishing it from existing reports in the literature.

Overall, µF-P10 displayed the most favorable balance of morphology, release behavior, and biological performance, surpassing both µF-P10T1 and the commercial dermal matrix. Nevertheless, several limitations must be acknowledged:

Extract incorporation was not directly detectable by FTIR, limiting confirmation of homogeneous distribution.

In vitro assays cannot fully replicate the in vivo periodontal microenvironment.

The release profile in PBS may not reflect enzymatic degradation and fluid dynamics in vivo [24,25].

## 4. Materials and Methods

### 4.1. Materials

This experimental study aimed to develop and characterize a composite polymeric scaffold incorporating a bioactive additive, specifically *T. vulgare* extract, with potential application as a substitute for subepithelial connective tissue grafts.

Polycaprolactone (PCL; Mw = 80,000 g/mol) was purchased from Sigma-Aldrich (St. Louis, MO, USA) and used as the primary polymeric matrix. The organic solvents chloroform (CHCl_3_), dichloromethane (DCM), and methanol (MeOH) (analytical grade) were obtained from Merck (Rahway, NJ, USA) and used to prepare electrospinning solutions. The bioactive additive, *Triticum vulgare* extract (*T. vulgare*), was purchased from Natural Green (Bogotá, Colombia). The extract was supplied as a liquid formulation in polyethylene glycol with a concentration ≤ 20%. The product was stored at room temperature and used without further purification. A commercial acellular dermal matrix (Puros^®^ Dermis Allograft Tissue Matrix, Zimmer Biomet, Palm Beach Gardens, FL, USA) was included as a reference control.

Phosphate-buffered saline (PBS, pH 7.4) was prepared in-house using sodium chloride (NaCl), potassium chloride (KCl), disodium phosphate (Na_2_HPO_4_), and monopotassium phosphate (KH_2_PO_4_), all analytical grade (Merck, Rahway, NJ, USA, and AppliChem Panreac, Darmstadt, Germany), dissolved in distilled water and adjusted to the target pH.

For cell culture experiments, Dulbecco’s Modified Eagle Medium (DMEM), fetal bovine serum (FBS), and penicillin–streptomycin (10,000 U/mL) were obtained from Thermo Fisher Scientific (Waltham, MA, USA). AlamarBlue^®^ reagent for viability assays was purchased from Thermo Fisher Scientific, and the Cell-Quant™ No Wash Cell Proliferation Assay Kit (Catalog No. A014) was obtained from BioVision (Milpitas, CA, USA). Triton™ X-100 was purchased from Sigma-Aldrich and used as a positive control for cytotoxicity.

### 4.2. Polymeric Solution Preparation and Membrane Preparation

Two polymeric matrices were prepared: the polymer base and the one loaded with the natural extract as a bioactive component, called μF-P10 and μF-P10T1 in an analogous way, respectively.

The base PCL solution was prepared by dissolving 10% (*w*/*v*) of the polymer in a 10 mL organic solvent mixture composed of chloroform, dichloromethane, and methanol in a 60:30:10 ratio, under continuous stirring for 1 h (Table 2). The ternary solvent system was selected based on compatibility with the Hansen solubility parameters of PCL (Table S1 in Supporting Information). Chloroform and dichloromethane provide efficient dissolution of the polymer backbone, while methanol increases the polarity and electrical conductivity of the solution, improving electrohydrodynamic jet stretching and fiber formation.

In the formulation with *T. vulgare*, 1 mL of extract was added directly, under constant stirring for 30 min.

Both scaffolds were fabricated using a QUBITeXp electrospinning unit under controlled conditions, with a voltage of 9.9 kV and a flow rate of 6.5 mL/h. The fabrication conditions included the use of an 18-G needle and a working distance of 12 cm. Fibers were deposited on a flat collector plate, forming three layers, with each layer spun for 10 min to complete the wrapping process. Each membrane was dried at 37 °C for 8 h to evaporate possible solvent residues.

#### Rheological Characterization of Spinning Solutions

The rheological behavior of the PCL (μF-P10) and PCL-TVE (μF-P10T1) solutions was characterized using a modular compact rheometer (MCR 92, Anton Paar, Graz, Austria). Measurements were performed at a controlled temperature of 25 °C. A cone-and-plate geometry (CP50-1, 50 mm diameter, 1° cone angle) was used to ensure a uniform shear rate throughout the sample. The dynamic viscosity was recorded as a function of the shear rate, ranging from 0.1 to 100 s^−1^. This range was selected to simulate the different flow regimes during the Taylor cone formation and jet ejection. All measurements were performed in triplicate to ensure reproducibility, and the average viscosity values at specific shear rates were used for subsequent analysis of the electrospinning stability.

### 4.3. Morphological Characterization of Membranes

The morphology of the electrospun membranes was evaluated by scanning electron microscopy (SEM) to determine the fiber diameter distribution, surface porosity, and structural integrity. A JEOL JSM-6460LV reference SEM microscope with 20 kV voltage was used (JEOL, Tokyo, Japan). The membranes were supported on carbon tape and sputter-coated with a gold layer to improve the conductivity.

To evaluate fiber diameter, the images were processed in ImageJ 1.54p (National Institutes of Health, Bethesda, MD, USA) to measure the average fiber diameter and its size distribution. In addition, surface porosity was quantified by observing the openings in the fiber mesh formed on the surface. The SEM images were analyzed using Python 3.13.2 software (Python Software Foundation, Wilmington, DE, USA). The membranes were evaluated at three random points on each sample to obtain representative results and compared with the characteristics of a commercial acellular dermal matrix as a control.

The total porosity of the electrospun membranes was determined using the liquid displacement method. Rectangular samples $1\times 4\;{\mathrm{cm}}^{2}$ were immersed in a known volume V=10 of distilled water. The samples remained submerged until saturation was reached. The total volume with the sample $V_{2}$ and the residual volume after removing the sample $V_{3}$ were recorded. The porosity percentage was calculated according to the displacement formula, providing a volumetric assessment of the internal void space, as requested by the peer review process.

### 4.4. Mechanical Properties of Membranes

The mechanical properties of the electrospun polymeric fibers and the acellular dermal matrix were evaluated using a texture analyzer (TA.XT Plus, Stable Micro Systems, Godalming, UK) operating in tensile mode. Before testing, the samples were briefly immersed in deionized water to ensure hydration and avoid brittleness during manipulation. Each specimen was positioned to maintain a tension zone approximately 3 cm long. Two different fixation methods were used to secure the samples to the equipment: directly between the grips of the texture analyzer and with a 5-0 silk suture (Demetech, Miami Lakes, FL, USA), which allowed assessment of tear resistance under conditions simulating surgical suture application. The tests were conducted under the following conditions: an initial force of 0.003 N was applied, with distance set as the variable along the X-axis. The extension distance was configured to 40 mm, and the crosshead speed was maintained at 0.1 mm/s throughout the experiment. During the assay, the equipment recorded the force until rupture, generating force–distance data.

The Young’s modulus of the membranes (1.0 cm × 5.0 cm) was determined in wet conditions (using purified water) using a Lamy Tx 700 texturometer (Lamy Rheology, Rue des Aulnes, France). A 50 N load cell and a displacement speed of 0.1 mm/s were used. The Young’s modulus was determined from the slope of the stress vs. strain curve. Five replicates were performed. The values obtained from the membranes loaded and unloaded with the plant extract were compared with a commercial product of decellularized human dermis.

### 4.5. Physicochemical Properties of Membranes

Fourier-transform infrared spectroscopy (FTIR) was used to characterize the physicochemical properties of the membranes. This technique was employed to visualize functional groups in the membrane structure and to investigate potential interactions between the base polymer (PCL) and the bioactive extract from *T. vulgare*. FTIR analysis was performed using a JASCO-6600 spectrophotometer (JASCO Inc., Tokyo, Japan) in the range of 4000 to 400 cm^−1^ with a resolution of 4 cm^−1^.

### 4.6. Quantification of T. vulgare Extract Released from Membranes Using Ultra-High-Performance Liquid Chromatography (UHPLC-MS)

A controlled experimental setup was used to evaluate the release kinetics of the active substance. A membrane section, 0.6 mm in diameter, loaded with *T. vulgare* extract, was placed in an Eppendorf tube containing 1 mL of phosphate-buffered saline (PBS, pH 7.4). The system was subjected to orbital shaking at 100 rpm and kept at a constant temperature of 37 °C. At predetermined time intervals (1 h, 2 h, 4 h, 6 h, 8 h, 24 h, and 48 h), 100 µL aliquots of the medium were taken for later analysis.

To determine the chemical profile and quantify the compounds released at each interval, each fraction of *T. vulgare* was analyzed by UHPLC-MS, using a Shimadzu Prominence LC (Shimadzu Corporation, Tokyo, Japan) equipped with a PDA detector and an LC-MS2020 mass spectrometer with a quadrupole analyzer and a DUIS interface. The column used was a Phenomenex (Torrance, CA, USA) Luna C18 (5 µm, 150 mm × 2 mm. The flow rate was 1 mL/min, and the mobile phases A and B were formic acid in water (0.1%) and formic acid in acetonitrile (0.1%), respectively. The gradient started at 5% B (0 min) and was maintained for 2 min. Then, B increased from 5% to 100% over 5 to 30 min and was maintained for 5 min. The oven temperature was 40 °C, and the wavelengths were 254 and 280 nm. The ESI interface was operated in positive ion mode with a capillary voltage of 4.5 kV and an endplate offset of 0.5 kV. The pressure of the nebulization gas was 0.4 Bar; the drying gas was maintained at a flow rate of 8 L/min at 200 °C. The collision and the quadrupole energy were set to 12 and 6 eV, respectively. RF1 and RF2 funnels were programmed to 150 and 200 Vpp, respectively. Finally, to calculate the concentration of *T. vulgare* samples at different release times, a calibration curve was constructed using the *T. vulgare* extract, enriched mainly in ferulic acid (*m*/*z* = 194.0579), at concentrations ranging from 10 to 100 mg/mL. Finally, the percentage of cumulative release was calculated as a function of time, as follows:

(1) $$\mathrm{The cumulative amount of release}\%=\frac{Ct}{C\infty }\times 100$$

where *Ct* is the *T. vulgare* extract amount released at time t, while C∞ is the initial *T. vulgare* extract amount. The results represent the mean ± SD of independent triplicate measurements.

### 4.7. General Cell Culture Conditions

Primary human gingival fibroblasts (FGH) were cultured in Dulbecco’s Modified Eagle Medium (DMEM) supplemented with 10% fetal bovine serum (FBS) and 1% penicillin–streptomycin under standard conditions (37 °C, 5% CO_2_, and humidified atmosphere). Cells were used at passages 17–20 and seeded at the densities specified in each experimental section.

### 4.8. Cell Viability Assay

#### 4.8.1. Stimulation of FGH with Membranes Loaded with *T. vulgare*

FGH were seeded at a density of 30,000 cells per well in 96-well plates. Upon reaching approximately 80% confluence, the culture medium was replaced, and the cells were stimulated with electrospun test membranes. The treatments consisted of membranes without bioactive compounds (µF-P10) and membranes loaded with *T. vulgare* extract (µF-P10T1). All membranes were cut into 6 mm diameter circular disks and sterilized by exposure to UV light for 15 min. Disks were carefully transferred into each well using sterile forceps. The stimulation periods were 24 and 48 h. Untreated cells served as a negative control, and a 1% Triton X-100 solution was used as a positive cytotoxicity control.

#### 4.8.2. Cell Viability Assay

Cell viability was assessed using the alamarBlue assay (Thermo Fisher Scientific, USA), based on the metabolic reduction in resazurin to the fluorescent compound resorufin by viable cells.

After the 24 and 48 h stimulation periods, the membranes and medium were removed from each well. Cells were gently washed once with 200 µL of phosphate-buffered saline (PBS), and the PBS was subsequently aspirated.

A working solution of resazurin was prepared by diluting 800 µL of resazurin stock into 7200 µL of non-supplemented DMEM. Then, 200 µL of this resazurin working solution was added to each well, and plates were incubated at 37 °C for 4 h in the dark.

Fluorescence was measured using a microplate reader (TECAN Infinite 200 PRO, TECAN, Männedorf, Switzerland) at excitation and emission wavelengths of 530 nm and 590 nm, respectively. Cell viability was expressed as a percentage relative to untreated control wells. All experiments were performed in triplicate for each treatment. Data were analyzed using GraphPad Prism version 6.0 (GraphPad Software Inc., San Diego, CA, USA).

(2) $$\mathrm{Cytotoxicity}\left(\%\right)=\frac{\left(\mathrm{Abs sample}-\mathrm{Control low}\right)}{\left(\mathrm{Abs Control High}-\mathrm{Control low}\right)}\times 100$$

### 4.9. Cell Proliferation Assay

Cell proliferation was evaluated in FGH after 48 h of stimulation with the membranes µF-P10 and µF-P10T1, as well as a commercial dermal matrix (Dermis). Cells were seeded at a density of 5000 cells per well in 96-well plates. A 4 h delay was allowed for cell adhesion before treatments were applied. Control groups included untreated cells and a positive control treated with 1% Triton X-100.

After 48 h, cell proliferation was assessed using the Cell-Quant No Wash Cell Proliferation Assay Kit (Catalog Number: A014) according to the manufacturer’s protocol. Fluorescence was measured at an excitation wavelength of 485 nm and an emission wavelength of 530 nm using a plate reader (TECAN Infinite 200 PRO, Switzerland). Proliferation was expressed relative to a calibration curve generated using cell densities of 1250, 2500, 5000, 10,000, 20,000, and 40,000 cells per well. All conditions were tested in six replicates for each treatment, except for the Dermis group, which was tested in four replicates.

### 4.10. Evaluation of Wound Closure in Response to Membrane Stimulation

#### 4.10.1. Cell Culture and Wound Induction

FGH cells were seeded at a density of 50,000 cells per well in 24-well plates and cultured until they reached approximately 90% confluence. A linear scratch wound was then created across the monolayer in each well using a sterile 200 µL pipette tip. Wells were gently washed with phosphate-buffered saline (PBS) to remove detached cells.

#### 4.10.2. Treatment Application

After wound induction, a 1 cm diameter membrane corresponding to each treatment group was applied directly onto the wounded area and maintained in contact with the fibroblast monolayer for 48 h. The treatment groups included a control group with no membrane as a positive control, a µF-P10 membrane without bioactive compounds, and a µF-P10T1 membrane loaded with *T. vulgare* extract and commercial Dermis (Puros^®^ Dermis Allograft Tissue Matrix).

#### 4.10.3. Evaluation of Wound Closure

Wound closure was evaluated by capturing images of the wound area using an inverted microscope (20× magnification) at two time points: immediately after treatment application (*t*_0_) and 48 h later. The same site was imaged at both time points for consistency.

The wound closure percentage was calculated using ImageJ 1.54p software (National Institutes of Health, Bethesda, MD, USA) by comparing the wound area at *t*_0_ with that after 48 h. The results were expressed as the percentage of wound closure relative to the initial wound area. All conditions were tested in triplicate to ensure reproducibility.

## 5. Conclusions

In this study, we successfully fabricated biomimetic PCL-based scaffolds loaded with *Triticum vulgare* extract (TVE) using an optimized ternary solvent electrospinning process. The integration of TVE significantly altered the rheological profile of the solution, reducing the zero-shear viscosity from 1750 mPa·s to 1200 mPa·s, which promoted the formation of a hierarchical, bimodal fiber architecture.

The resulting μF-P10T1 membranes exhibited a volumetric porosity of 42.68% and a Young’s modulus of 3.54 MPa, providing a robust mechanical environment that matches the requirements for subepithelial connective tissue regeneration.

Biologically, the loaded scaffolds demonstrated superior performance by enhancing human gingival fibroblast (HGF) proliferation and maintaining cellular viability through the sustained release of antioxidant compounds. These findings suggest that the μF-P10T1 scaffold is a promising, bioactive alternative to autogenous grafting, potentially reducing surgical morbidity in the treatment of gingival recession.

Future perspectives should focus on long-term in vivo validation to assess degradation kinetics and the scaffold’s integration within the complex periodontal microenvironment, with the goal of eventual clinical translation as a standardized, off-the-shelf substitute.

## Figures and Tables

**Table 1:** Rheological properties and flow behavior of PCL and PCL-TVE spinning solutions at 25 °C.

Solution	TVE (%)	Zero-Shear Viscosity (at 0.1 s^−1^)	High-Shear Viscosity (at 100 s^−1^)
μF-P10	0	1750 mPa·s	700 mPa·s
μF-P10T1	1	1200 mPa·s	600 mPa·s

**Table 2:** The parameters for creating nanofibers using electrospinning processes.

No.	Membrane	PCL (% *w*/*v*)	*T. vulgare* (mL)	Fill to Capacity (mL)	Voltage (kV)	Flow Rate (mL/h)	Total Time (min)
1	μF-P10	10	0	10	9.9	6.5	30
2	μF-P10T1	10	1	10	9.9	6.5	30

## Data Availability

The data supporting the findings of this study are not publicly available due to ethical restrictions and the protection of confidential information related to potential future patents. Data may be available from the corresponding author upon reasonable request, subject to appropriate confidentiality agreements.

## Supplementary Materials

The following supporting information can be downloaded at: https://www.mdpi.com/article/10.3390/molecules31091505/s1, Table S1: Hansen solubility parameters and thermodynamic distance calculation for the PCL/ternary solvent system; Figure S1: Flow curves and viscosity characterization of the control PCL solution.; Figure S2: Flow curves and viscosity characterization of the control PCL with *T. vulgare* extrac solution.
