# Supplementary material for: Electrospun Fibers Encapsulating Triticum vulgare Extract as a Potential Scaffold for the Regeneration of Subepithelial Connective Tissue
Source: Molecules. 2026 May 1;31(9):1505. doi: 10.3390/molecules31091505 (PMC13165391; doi:10.3390/molecules31091505)
Supplement: Supplementary file 1 [file molecules-31-01505-s001.zip › molecules-4154061-supplementary.pdf]

Supporting Information

Physicochemical and Thermodynamic Rationale for the Selection of the Ternary Solvent System

1. Introduction

The selection of the solvent system in electrospinning is a critical parameter that governs solution properties, jet stability, and ultimately fiber morphology. In this study, a ternary solvent system composed of chloroform ($CHCl_3$), dichloromethane (DCM), and methanol (MeOH) in a 60:30:10 volume ratio was employed to modulate the physicochemical properties of the polycaprolactone (PCL) solution and promote the formation of hierarchical fibrous structures resembling the extracellular matrix (ECM).

This section provides a thermodynamic and transport-based rationale for the selection of this solvent mixture.

2. Hansen Solubility Parameter Analysis

The total solubility parameter is defined as:

$$\delta_t^2 = \delta_d^2 + \delta_p^2 + \delta_h^2$$

where:
δ_d = dispersion interactions
δ_p = polar interactions
δ_h = hydrogen bonding

The Hansen distance between polymer and solvent is given by:

$$R_a = sqrt\left(4 * \left(\delta_d^p - \delta_d^s\right)^2 + \left(\delta_p^p - \delta_p^s\right)^2 + \left(\delta_h^p - \delta_h^s\right)^2 \right)$$

For good solubility:

$$R_a < R_0$$

Material	δd	δp	δh
**PCL**	17.0	4.8	8.3
**$CHCl_3$**	17.8	3.1	5.7
**DCM**	18.2	6.3	6.1
**MeOH**	15.1	12.3	22.3

3. Solvent Evaporation and Peclet Number

The solvents present significantly different vapor pressures and boiling points:

Solvent	Boiling Point (°C)	Vapor Pressure
**$CHCl_3$**	61	High
**DCM**	40	Very high
**MeOH**	65	Moderate

$$Pe = (R * v) / D$$

where:
R = jet radius
v = jet velocity
D = solvent diffusion coefficient

When:

$$Pe >> 1$$

Evaporation dominates over diffusion, leading to the formation of a solid shell and a solvent-rich core.

4. Electrohydrodynamic Scaling

The electrical force per unit volume is:

$$f_e = \rho_e * E$$

where:
ρ_e = charge density
E = electric field

The electric field is defined as:

$$E = \frac{V}{d}$$

where:
V = applied voltage
d = tip-to-collector distance

The scaling law for fiber diameter is:

$$d\_f \sim \left(\frac{(\eta * Q)}{\varepsilon_0 * E^2}\right)^{\frac{1}{3}}$$

where:
d_f = fiber diameter
η = viscosity
$Q = flow\ rate$
ε_0 =vacuum permittivity

6. Mass Transport in Fibrous Structures

The effective diffusivity in porous media is:

$$D_{eff} = D_0 * (\varepsilon/\tau)$$

where:
D_0 = diffusion coefficient in free medium
ε = porosity
τ = tortuosity

7. Key Interpretation

The combination of:

- solvent evaporation rate
- solution viscosity
- electrical forces

governs jet stability and fiber morphology, enabling the formation of hierarchical structures.

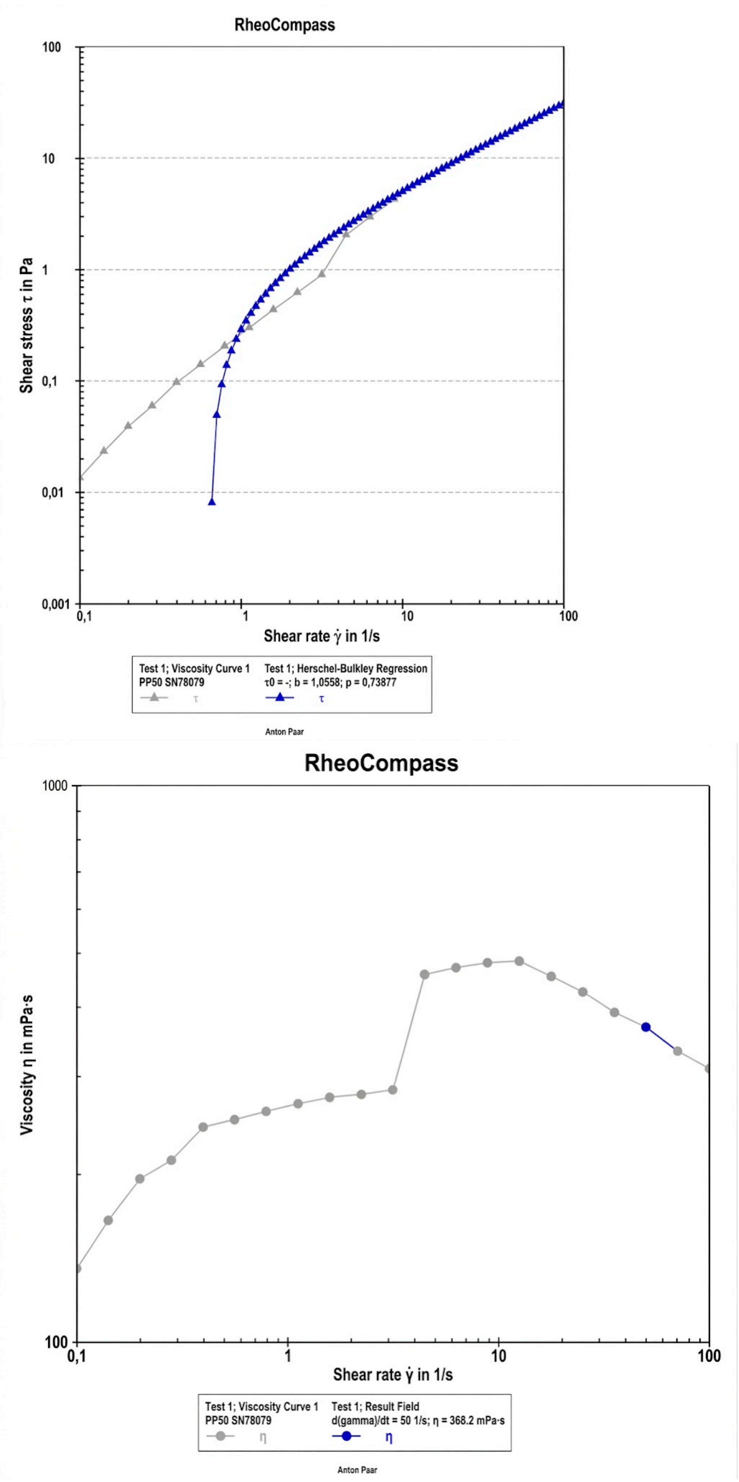

Figure S1. Dynamic viscosity as a function of shear rate for the neat PCL solution (**μF-P10**), showing a pseudoplastic behavior characterized by a zero-shear viscosity of approximately 1750 mPa s.

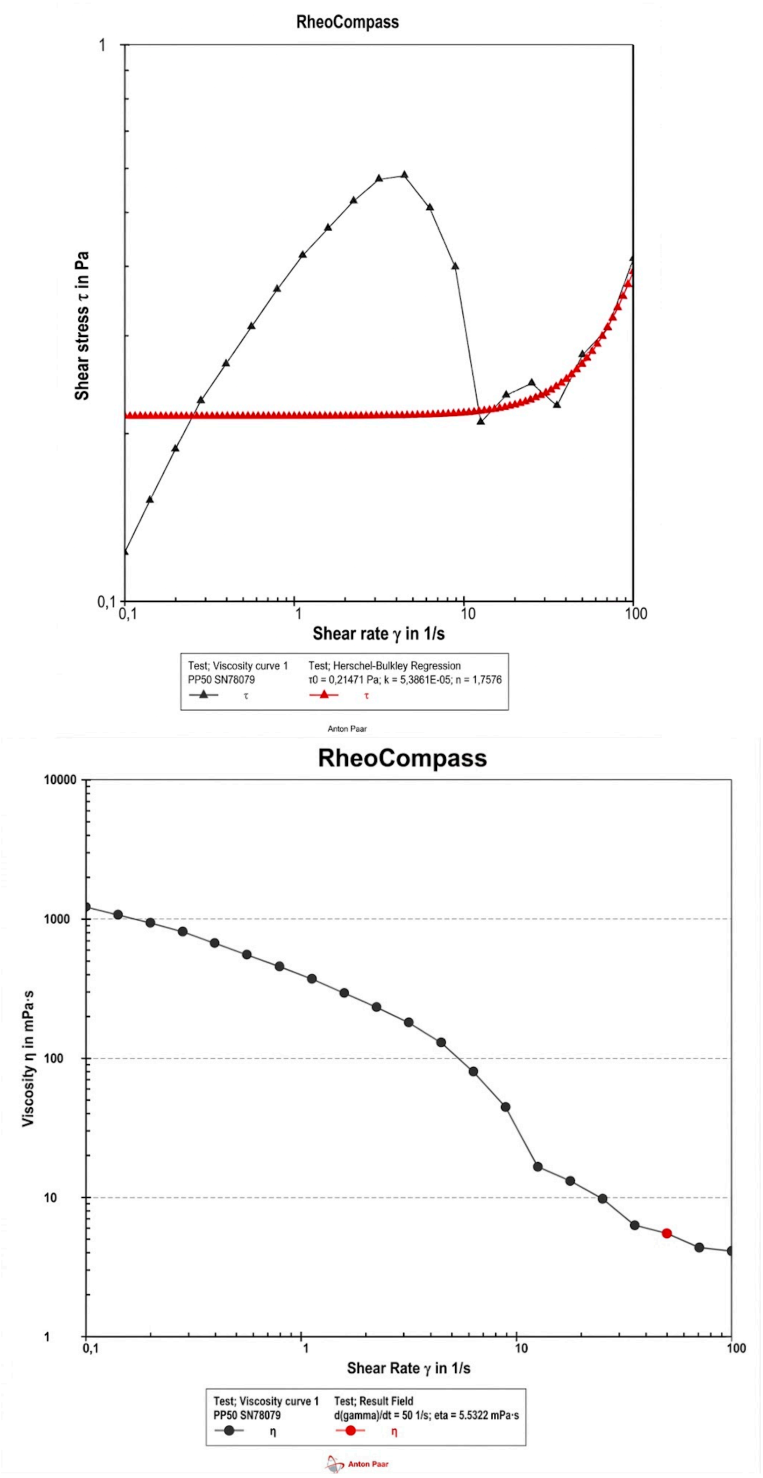

Figure S2. Rheological profile of the PCL solution loaded with *Triticum vulgare* extract (μF-P10T1). The incorporation of TVE induced a reduction in zero-shear viscosity to approximately 1200 mPa s, suggesting a plasticizing effect of the phytotherapeutic compounds.
